# Diagnosis, Treatment, and Current Controversies in Multiligament Knee Injuries: A Narrative Review

**DOI:** 10.7759/cureus.116740

**Published:** 2026-09-22

**Authors:** Max A Saráchaga Mendoza

**Affiliations:** 1 Orthopedic Surgery, Hospital General de México "Dr. Eduardo Liceaga", Mexico City, MEX

**Keywords:** graft selection, knee arthrofibrosis, knee dislocation, ligament reconstruction, multiligament knee injury, posterolateral corner, schenck classification, surgical timing

## Abstract

Multiligament knee injuries are infrequent but potentially severe, defined by the simultaneous rupture of two or more major knee ligaments, with a considerable risk of neurovascular injury. This narrative review, based on a search of PubMed, the Cochrane Library, and Google Scholar covering 2010 to 2026, examines their diagnosis, treatment, and outcomes, with emphasis on current controversies in surgical management. The Schenck classification remains the reference system, but its initial category is imprecise, and it does not reliably predict neurovascular injury; it should be interpreted alongside the full injury profile. Diagnosis requires a high index of suspicion and systematic physical examination; magnetic resonance imaging is the diagnostic standard, with a low threshold for computed tomography angiography when vascular injury is suspected. Surgery is the treatment of choice in active patients. Single-stage anatomic reconstruction of all injured structures within the first two to three weeks is associated with less secondary meniscal and chondral injury. Autograft is preferred for the anterior cruciate ligament in young patients, whereas autograft and allograft perform comparably for the posterior cruciate ligament and collateral complexes. Early mobilization is associated with less arthrofibrosis without compromising the grafts. Functional outcomes are acceptable in the short and medium term but deteriorate from seven years onward, with post-traumatic osteoarthritis in up to 71.7% of patients in some series. This should be discussed in preoperative counseling. Arthrofibrosis and residual instability are the most frequent complications, and neurovascular injury is the most severe. Surgical timing, graft selection, and the sequence of reconstruction remain the main open questions and will require comparative studies.

## Introduction and background

Multiligament knee injuries (MLKIs) involve the simultaneous disruption of two or more of the four main ligamentous structures of the knee: the anterior cruciate ligament (ACL), the posterior cruciate ligament (PCL), the medial collateral ligament (MCL), and the lateral collateral ligament (LCL) [1-3]. Their most severe form is knee dislocation, that is, the complete loss of femorotibial joint congruity at the moment of trauma, although a substantial proportion of patients present with spontaneous reduction before the initial assessment [1,4]. For this reason, an MLKI is now considered a functional diagnosis, based on documented ligamentous disruption rather than exclusively on radiographic findings [1,4].

MLKIs are uncommon but severe: they compromise joint stability and the neurovascular viability of the affected limb. They predominate in young male patients and are usually associated with high-energy mechanisms, although in recent years a low-energy pattern has been described in patients with obesity, together with a growing volume of sports-related cases in young, athletic populations [4,5].

The associated injuries, mainly vascular and nerve lesions, largely determine the severity of the injury. Many patients also present with concomitant meniscal injuries, and a notable percentage are associated with periarticular fractures [1,6,7]. The 1994 Schenck classification remains the reference system in clinical practice and in the literature; recent revisions have proposed incorporating open injuries and extensor mechanism disruption, and reassessing its prognostic value [8-11].

The treatment of these injuries has changed markedly over the past two decades. The available evidence supports surgical treatment as the first option in active patients: expert consensus is unanimous on this point, and the functional outcomes and satisfaction rates reported after reconstruction exceed those of historical series managed conservatively, although no contemporary series includes a conservatively managed comparison group [2,3]. Nevertheless, controversies persist in several aspects of management: the optimal timing of surgery (early versus delayed reconstruction), the choice between ligament repair and reconstruction, the decision between autograft and allograft, and the optimal sequence of reconstruction of the injured structures. The most recent systematic reviews show heterogeneous results and do not establish a clear superiority of any single strategy [12-14].

Long-term outcomes remain suboptimal: the function recovered at two years deteriorates progressively thereafter, more so in injuries involving the PCL, and post-traumatic osteoarthritis and arthrofibrosis are the main complications [15-17].

The evidence base is limited. Most studies are retrospective case series with small samples, randomized comparative trials are scarce, and methodological heterogeneity impedes quantitative synthesis. The first international Delphi consensus provided a common framework on nomenclature, diagnosis, treatment, and rehabilitation, and acknowledged the need for further research [2].

Surgical technique and rehabilitation protocols have changed substantially in recent years, but no recent synthesis integrates the biomechanical rationale, the contemporary revisions of the Schenck classification, and the evidence on timing, graft choice, reconstruction sequence, rehabilitation, and long-term outcomes in a single account oriented toward surgical decision-making [2,3]. The objective of this review is to fill that gap by examining the diagnosis, classification, surgical treatment, rehabilitation, and long-term outcomes of MLKIs, and by identifying which controversies over timing, graft selection, and reconstruction sequence remain unresolved and what evidence is needed to settle them.

## Review

Search strategy

This narrative review of MLKIs is based on a search of PubMed (the main source, since it covers the orthopedic and trauma literature), the Cochrane Library (systematic reviews and controlled clinical trials), and Google Scholar (Spanish-language publications and classic reference articles). The search covered 2010-2026, plus a few earlier articles of historical or conceptual value: the original Schenck classification (1994) and the knee dislocation series by Kennedy (1963) and Meyers and Harvey (1971) [8,18,19]. The most recent search was performed on September 14, 2026. Medical Subject Headings (MeSH) and free-text terms were combined with the Boolean operators AND and OR in five strings: (1) "multiligament knee injury" OR "knee dislocation" OR "multiple ligament knee reconstruction"; (2) "anterior cruciate ligament" AND "posterior cruciate ligament" AND ("posterolateral corner" OR "posteromedial corner"); (3) "multiligament knee" AND ("timing" OR "early repair" OR "delayed reconstruction" OR "graft" OR "allograft" OR "autograft"); (4) "Schenck classification" OR "knee dislocation classification" OR "KD classification knee"; and (5) "multiligament knee" AND ("functional outcome" OR "IKDC" OR "Lysholm" OR "KOOS" OR "complication" OR "neurovascular injury").

A MLKI was defined as disruption of two or more of the four main ligamentous structures (ACL, PCL, medial complex, and lateral complex), whether or not a dislocation was radiographically documented; dislocations with fewer than two injured structures were not eligible. Adult was defined as 16 years or older. Studies combining MLKIs with isolated single-ligament injuries were eligible only when the multiligament data could be identified separately. Eligible articles were written in English or Spanish, published in that period, and addressed diagnosis, classification, surgical treatment, or functional outcomes; eligible designs were systematic reviews, meta-analyses, clinical trials, cohort studies, case series with a relevant sample size, and clinical practice guidelines. Excluded were patients younger than 16 years; isolated case reports, letters, or opinion pieces without bibliographic support; isolated single-ligament injuries without a multiligament component; and articles without full-text access. Duplicates were removed by comparing author, title, journal, and year; screening by title and abstract followed by full-text review of the preselected articles yielded the 67 articles that constitute the body of this review. No specific Cochrane review on MLKI management was identified. The synthesis is narrative: no pooled estimate, heterogeneity statistic, or meta-regression was computed, and the effect estimates cited are reproduced from and attributed to their source studies rather than combined.

Epidemiology, anatomy, and biomechanics

Epidemiology and Injury Mechanisms

Knee dislocations, the most severe expression of the MLKI spectrum, account for 0.02% to 0.2% of all trauma injuries managed [1,4,20]. This figure probably underestimates the true incidence, because up to 50% of tibiofemoral dislocations reduce spontaneously before hospital assessment, which hinders their recognition on the initial plain radiograph and requires the diagnosis to be based on ligamentous instability documented by physical examination and magnetic resonance imaging (MRI) [4,20,21].

Regarding the patient profile, men predominate (52% to 65% of cases), with a mean age close to 35 years [4,20]. Classic series describe a bimodal age distribution: a first peak in young, active adults secondary to sports and high-energy accidents, and a second peak in older adults related to low-energy falls in patients who are overweight or obese [4].

Injury mechanisms are usually classified into three groups according to the energy of the trauma. High-energy mechanisms, such as motor vehicle accidents (particularly motorcycles, which account for up to 52% of cases in some series), falls from height, industrial trauma, and high-impact sports trauma, are the most frequently described in the classic literature and are associated with a higher frequency of neurovascular injuries and periarticular fractures [1,4,20]. Low-energy mechanisms, associated with pivoting and contact sports, are increasingly recognized in young, athletic populations [5]. Finally, an ultra-low-energy pattern has been described, such as falls from standing height or twisting injuries, usually in patients with morbid obesity. Although the mechanism is mild, the injuries tend to be severe and may be accompanied by neurovascular complications [1,4,22].

Each mechanism produces a different injury pattern, with different structural involvement, risk of associated injuries, and prognosis. Dean et al. found better postoperative activity scores after low-energy than after high-energy MLKIs, so the mechanism behaves as an independent prognostic factor [23].

Anatomy of the Main Ligamentous Structures

Planning an anatomic reconstruction requires precise knowledge of the anatomy of the four main ligamentous stabilizers of the knee. The ACL is the primary static stabilizer of anterior tibial translation. It is composed of two functional bundles - the anteromedial and the posterolateral - which tension at different flexion angles. The anteromedial bundle is the main restraint to anterior translation in flexion; the posterolateral bundle takes over in extension and also helps control tibial rotation [3].

The PCL is the primary static stabilizer of posterior tibial translation and, together with the lateral structures, forms a functional unit that maintains normal knee kinematics. It is composed of two main bundles: the anterolateral, which is bulky and tightens in flexion, and the posteromedial, which tightens in extension. Recognition of this anatomy led to double-bundle reconstruction techniques, which have shown more physiological biomechanical results in cadaveric and clinical studies [24,25].

The posteromedial corner (PMC) is formed by the superficial MCL, the deep MCL, the posterior oblique ligament, the oblique popliteal ligament, and the posteromedial capsule. Its combined function includes resistance to valgus stress and rotational stabilization in flexion, and the oblique popliteal ligament, an expansion of the semimembranosus across the posterior capsule, contributes with the posteromedial and posterolateral capsule to restraining hyperextension [26,27]. The superficial MCL stabilizes rotational kinematics, an effect that is amplified in combined injuries with the ACL [26].

The posterolateral corner (PLC) is functionally more complex than its medial counterpart. Its three principal static stabilizers, the LCL, the popliteus tendon, and the popliteofibular ligament, have different biomechanical roles. The LCL is the main passive restraint to varus loads, with maximal efficacy between full extension and 30° of flexion; in greater arcs, its contribution decreases. It also contributes secondarily to restraining internal and external tibial rotation [3,5]. PLC involvement is an independent risk factor for failure of ACL reconstruction when it is not addressed surgically, since residual rotational instability can overload the graft [28].

Biomechanics of the Knee With a Multiligament Injury

The joint biomechanics of a knee with a MLKI differs substantially from that observed in isolated ligament injuries. Simultaneous disruption of two or more main stabilizers produces multidirectional instability that the remaining structures cannot compensate for. This overloads the articular cartilage and the menisci and predisposes to early degenerative changes.

Cadaveric biomechanical studies have clarified the relative role of each structure. Using the cadaveric heel-height test, Perry et al. showed that the ACL is the main passive restraint to knee hyperextension, with a secondary but substantial contribution from the PLC and the MCL [29]. The posterior capsule also contributes to this restraint: in a robotic study of 24 cadaveric knees, Noyes et al. found that the combined posteromedial and posterolateral capsular structures, together with the oblique popliteal ligament, provide the major resisting moment against abnormal hyperextension, whereas the cruciate ligaments act as secondary restraints [27]. The two studies load the joint differently, so their results are compatible. The posterior capsule should therefore be assessed in injuries produced by a hyperextension mechanism; the authors also emphasize the potential role of concomitant repair of the posterior capsular structures in multiligament injuries [27].

This functional interdependence explains why, in the presence of a combined injury, angular displacement and clinical instability can exceed the sum of the instabilities expected in isolated injuries. Likewise, Toyooka et al. described recognizable patterns of combined injuries in patients with PLC involvement, in which coexistence with PCL and MCL injuries reflects a sequential load transfer during the injury mechanism rather than chance [28].

A reconstruction that does not address all injured structures can leave residual instability that overloads the implanted grafts and accelerates joint degeneration. This principle is the basis of the current approach of comprehensive anatomic reconstruction of all injured structures, ideally in a single surgical stage when technically feasible [2,3,30].

Finally, MLKIs rarely present as a pure ligamentous injury. A high proportion of patients have concomitant meniscal injuries, with a predominance of radial tears of the lateral meniscus in injuries involving the PLC [7]. Systematic recognition of associated meniscal and chondral injuries, particularly during preoperative planning with MRI, avoids incomplete treatment.

Classification systems

Original Schenck Classification (1994)

The classification most widely used today to describe knee dislocations and, by extension, MLKIs was originally proposed by Schenck in 1994 based on the analysis of the injured ligamentous structures, regardless of the injury mechanism or the direction of the dislocation [8]. Compared with previous classifications, based on the relative position of the tibia with respect to the femur (anterior, posterior, medial, lateral, or rotational), the Schenck system centered the diagnosis on ligamentous integrity, which is especially useful in the substantial proportion of cases in which the dislocation reduces spontaneously before the initial assessment [9,31]. The classification establishes five grades of increasing severity defined by the number and type of injured ligamentous structures. Grade KD I designates multiligament injuries with at least one intact cruciate ligament. KD II corresponds to isolated injury of both cruciate ligaments without involvement of the collateral ligaments, an uncommon pattern that usually occurs through pure hyperextension mechanisms. When rupture of one of the collateral complexes is added to the injury of both cruciate ligaments, the injury is termed KD III, differentiating the medial subtype (KD III-M) from the lateral subtype (KD III-L); this grade is the most frequent finding in large series. Involvement of the four major ligamentous structures defines KD IV, and the concomitant presence of a periarticular fracture corresponds to grade KD V [1,9].

The classification also incorporates two modifiers: the letter C to denote injury of the popliteal artery and the letter N for nerve injury, generally of the common peroneal nerve [1,9]. It remains in use more than 30 years after its publication.

Modifiers and Contemporary Revisions

Several groups have proposed extensions for situations the original system did not contemplate. Using an international Delphi consensus, Held et al. proposed the incorporation of a specific modifier for open injuries, given that their prognosis differs substantially from that of equivalent closed injuries [10].

Through a similar Delphi process with a panel of 46 specialized surgeons, Medvecky et al. addressed the integration of extensor mechanism involvement into the classification system. Disruption of the extensor mechanism, whether through patellar tendon avulsion, quadriceps rupture, or patellar fracture, substantially alters both surgical planning and the rehabilitation protocol, so mentioning it explicitly within the classification refines the prognostic stratification [32].

In a systematic review of the KD V category, Marcel et al. found marked heterogeneity in the way authors describe fracture-dislocations. The authors conclude that the KD V category groups together very different pathological entities, ranging from tibial plateau avulsion fractures to comminuted distal femoral fractures, which hinders comparison across series and limits the prognostic usefulness of the grade taken in isolation. They therefore recommend specifying the underlying fracture pattern when describing a case as KD V [33].

Limitations and Current Debates

The internal validity of some categories is also debated. In a retrospective multicenter study of the KD I category, Green et al. found that many cases classified as such do not correspond to documented femorotibial dislocations [34]. The subsequent systematic review and meta-analysis by the same group confirmed this trend: a femorotibial dislocation is radiographically documented at the time of injury in fewer than 1% of cases published as KD I, which has led some authors to propose the elimination or redefinition of this category, or a subclassification according to whether the dislocation was radiographically confirmed [35].

A second debate concerns the prognostic value of the Schenck grade. In a multicenter study of 144 patients, Sanchez-Munoz et al. showed that the Schenck grade does not reliably predict the presence of associated vascular injury, although it does show a significant association with the risk of neurological injury [11]. This finding challenges the assumption that a higher grade implies a higher overall neurovascular risk, and supports assessing vascular status in every patient irrespective of the assigned category.

Schneebeli et al., studying functional outcome at two to eight years of follow-up, found that the Schenck grade alone has limited discriminative capacity relative to variables such as the presence of associated meniscal or chondral injuries, the injury mechanism, or the timing of surgery [36]. The Schenck classification remains useful for describing and communicating the injury pattern, but it cannot be interpreted in isolation: prognostic assessment requires the context of the associated injuries.

Clinical and imaging diagnosis

The diagnosis of MLKIs requires a high index of clinical suspicion, given that up to half of tibiofemoral dislocations reduce spontaneously before the initial medical assessment. In these cases, the knee may appear reduced and have apparently normal plain radiographs, which frequently leads to delayed or incomplete diagnoses [31]. The scoping review by Makaram et al., which included 417 studies, identified clinical assessment and imaging planning as persistent areas of controversy, with no universally accepted diagnostic standards [37].

Clinical History and Physical Examination

The history should accurately record the injury mechanism, the energy involved, the time elapsed since the trauma, and any neurovascular alteration documented at the time of initial care, since these factors directly determine the probability of associated injuries and the urgency of complementary examinations [31].

Physical examination should be performed systematically, compartment by compartment, beginning with inspection and palpation in search of obvious deformity, open wounds, ecchymosis, and the status of the extensor mechanism. The integrity of the ACL is assessed with the Lachman test and the anterior drawer. That of the PCL is assessed with the posterior drawer and the posterior sag test with the knee at 90° of flexion. Coronal stability is examined by applying valgus and varus stress at 0° and 30°, assessing the MCL and the LCL respectively. The dial test, performed at 30° and 90° bilaterally, indicates PLC involvement when the asymmetry exceeds 10° and is present only at 30°, or a more extensive injury with PCL involvement when it persists at 90° as well [4,31]. The presence of instability in multiple planes confirms the multiligament pattern [4,31]. The initial vascular examination includes assessment of distal pulses and the ankle-brachial index (ABI).

Imaging Assessment

Anteroposterior and lateral plain radiographs of the knee are the initial imaging examination. They allow identification of associated periarticular fractures, bony avulsions characteristic of corner injuries such as the Segond fracture or the avulsion fracture of the fibular head, and, in unreduced cases, femorotibial incongruity. Nevertheless, in spontaneously reduced dislocations, the radiograph may be normal, so clinical-radiographic correlation is necessary. Stress radiographs, using forced varus and valgus and the posterior translation test under fluoroscopy, reached 89.7% agreement in the international Delphi process by Murray et al., which considered them useful for the objective quantification of the degree of instability in each compartment [2].

MRI is the gold standard for assessing the ligamentous structures of the knee. It allows highly accurate identification of injuries of the ACL, PCL, MCL, LCL, and the PMC and PLC complexes, as well as concomitant meniscal, chondral, and bony injuries [38]. However, the sensitivity of MRI for detecting PMC and PLC injuries in the subacute or chronic setting can be as low as 48% compared with clinical examination, especially when the initial edema has resolved and the morphological changes are subtle [39]. In these cases, high-resolution musculoskeletal ultrasound is a useful complementary tool: it allows dynamic assessment during stress maneuvers and shows minor alterations such as thickening, hypoechogenicity, and loss of the fibrillar pattern that may go unnoticed on MRI [39]. Computed tomography (CT) does not provide direct information on soft tissues, but it is very useful in preoperative planning for the detection of periarticular fractures and the assessment of tunnel convergence in complex multiligament reconstructions [38].

When vascular injury is suspected, international consensus recommends a low threshold for performing CT angiography in the setting of high-energy trauma and in certain injury patterns, especially in the presence of injury to both cruciate ligaments and the PLC [2]. MR angiography is a valid alternative with the added advantage of providing simultaneous information on the ligamentous structures. Conventional arteriography, historically considered the gold standard for vascular diagnosis, has progressively been displaced by CT angiography, which is more accessible, less invasive, and faster in the emergency setting. The choice among these studies depends on the injury mechanism, the patient's hemodynamic status, and the available resources.

Associated neurovascular injuries

Neurovascular injuries are the most serious complication in MLKIs. A delayed diagnosis or deficient management can threaten the viability of a limb, and neurological sequelae may be permanent. The popliteal artery and the common peroneal nerve are usually the most affected structures, because both are tethered close to the joint: the artery between the adductor hiatus and the soleal arch and the nerve at the fibular neck. This anatomy makes them especially susceptible to traction and shear forces caused by femorotibial displacement [22].

Incidence and Prognostic Impact

Constantinescu et al. synthesized the evidence on neurovascular injury in 19 studies comprising more than 37,000 knee dislocations. Vascular injury affected approximately one in 10 knees (10.7%), and 62.2% of those cases required surgical repair. With early detection protocols, amputation was reduced to 2.2% overall. Neurological injury was more frequent, affecting 19.6% of cases, although with a highly variable prognosis depending on severity [6]. Gomez-Bermudez et al. placed the incidence of vascular injury between 7% and 48% depending on the series, and noted that up to one-third of patients may present with popliteal artery injury at the initial assessment, with particular concentration in posterior dislocations, where such injury can reach 44% of cases [40].

Delay in revascularization beyond eight hours from the trauma is associated with amputation rates close to 86%, in contrast to current figures ranging between 2% and 4% with modern diagnostic-therapeutic protocols [22,40]. This substantial reduction in amputation rates, from the 36% to 40% recorded in 2014 to the 3.8% reported in the United States in 2020, is attributed to advances in early detection and in interventional vascular surgery techniques [22]. The predominant pathophysiological mechanism is traction or direct transection of the popliteal artery during anteroposterior tibiofemoral translation, although compression, intimal tear, and secondary thrombosis also occur and may present in a delayed fashion, making close surveillance necessary even with initially preserved pulses.

Predictors of Neurovascular Injury

The multicenter study by Sanchez-Munoz et al., conducted on 144 patients with surgically treated MLKIs, identified vascular injury in 3.5% of cases and neurological injury in 11.8%, lower than the figures reported in the dislocation series above because the denominator is a surgically treated MLKI cohort rather than documented dislocations. None of the variables analyzed, including the Schenck grade, age, sex, and injury mechanism, was significantly associated with the presence of vascular injury, which questions the predictive validity of the current classification systems for this specific complication.

PLC injury was the strongest predictor of neurological damage in this analysis, although the confidence interval is so wide that the magnitude of the association cannot be established; patients with posterolateral involvement were more than 12 times as likely to sustain a common peroneal nerve injury as those without it (OR 12.66; 95% CI 1.63-100; p = 0.02). MCL injury, by contrast, was a protective factor, reducing the neurological risk to less than one-third (OR 0.29; 95% CI 0.10-0.87; p = 0.03), a finding the authors attribute to the different nerve traction vector according to the direction of femorotibial displacement. Finally, each additional Schenck grade multiplied the risk of neurological injury by 2.5 (OR 2.47; 95% CI 1.36-4.50; p = 0.003) [11].

Neurological injury predominantly affects the common peroneal nerve, whose intimate relationship with the PLC makes it especially vulnerable in dislocation patterns with posterolateral involvement, such as KD III-L and KD IV. Clinical manifestations include foot drop, hypoesthesia on the dorsum of the foot, and paresis of the ankle extensors, with a variable and often incomplete recovery prognosis. That prevalence includes injuries of varying severity and recovery potential [6].

Diagnostic and Management Protocol

The ABI is measured in every patient as part of the initial assessment, not only when vascular injury is suspected. A value below 0.9 indicates the need for an urgent vascular study by CT angiography or conventional arteriography [40]. In the presence of signs of arterial injury such as absent pulses, active ischemia, expanding hematoma, or a bruit, urgent surgical exploration should not be delayed pending radiological confirmation. The usual sequence is temporary stabilization of the knee with an external fixator, then vascular repair by direct suture or venous graft bypass, and definitive ligament reconstruction in a delayed stage. Prophylactic fasciotomy should be considered when ischemia times are prolonged or when an impending compartment syndrome is suspected [40]. Table 1 summarizes the assessment of these injuries stage by stage, from the emergency evaluation described here to rehabilitation, with the decision rule and the evidence behind each step.

**Table 1:** Stepwise management algorithm for multiligament knee injuries ABI: ankle-brachial index; AP: anteroposterior; CT: computed tomography; MRI: magnetic resonance imaging; OR: odds ratio; CI: confidence interval Compiled from the evidence cited in the corresponding sections of this review.

Stage	Action	Decision rule
1. Emergency assessment	Reduce the dislocation when present; document distal pulses and the ABI	Measure the ABI in every patient; a value below 0.9 calls for urgent CT angiography or arteriography. Absent pulses, active ischemia, expanding hematoma, or bruit: urgent surgical exploration without waiting for imaging [40]
2. Vascular management	Temporary external fixation, vascular repair, then delayed ligament reconstruction	Vascular injury in 10.7% of knees; delayed revascularization beyond eight hours is associated with amputation rates close to 86%. Consider fasciotomy with prolonged ischemia [6,22,40]
3. Neurological assessment	Examine common peroneal nerve function (foot drop, dorsal hypoesthesia, extensor paresis)	Heightened suspicion with posterolateral corner involvement (OR 12.66; 95% CI 1.63-100, a wide interval that fixes only the direction of the association) and with each additional Schenck grade (OR 2.47) [11]
4. Imaging	AP and lateral radiographs, then MRI; stress radiographs to quantify instability	MRI is the diagnostic standard; a normal radiograph does not exclude a spontaneously reduced dislocation [2,4,31]
5. Classification	Assign the Schenck grade with the C and N modifiers	Use for description and communication, not for prognosis or for predicting neurovascular injury [8-11]
6. Surgical planning	Single-stage anatomic reconstruction of all injured structures within the first two to three weeks	92.3% expert agreement on single-stage surgery whenever possible. Staged approach with vascular emergency, polytrauma, periarticular fracture, or soft-tissue compromise [2,6]
7. Graft selection	Choose by structure, patient age, activity level, and available tissue	See Graft Selection section
8. Rehabilitation	Early controlled mobilization from the first 24 to 48 postoperative hours, progressing by objective criteria	Four sequential phases; return to competitive sport usually requires nine to 12 months and objective functional testing

Surgical timing

The timing of intervention is one of the most debated topics in MLKI surgery. Three broad intervals are used: early or acute surgery, conventionally defined as that performed within the first three weeks after the injury; delayed or subacute surgery, between three weeks and three months; and late surgery, beyond three months. These definitions show notable heterogeneity across studies, with cutoff points ranging from 48 hours to one year to delimit early surgery, which limits the synthesis and comparison of results [41].

Historical Evolution and Current Arguments

Historically, immediate surgical intervention by open repair followed by cast immobilization was the standard treatment, but it was associated with high rates of stiffness and arthrofibrosis, which led to delayed treatment as a strategy to reduce this complication. Arthrofibrosis, defined as the excessive formation of scar tissue with limitation of joint mobility, remains one of the most frequent complications of multiligament reconstructions [15]. With the generalization of arthroscopic techniques and accelerated rehabilitation protocols, this risk has decreased substantially, which has revived interest in early surgery.

The arguments in favor of early treatment include the possibility of taking advantage of the window of tissue reparability before scar reorganization, the reduction in the risk of secondary meniscal and chondral injuries associated with persistent instability, the prevention of disuse muscle atrophy, and the reduction in time away from work and sport. The arguments in favor of delayed treatment are based on the improvement of preoperative range of motion after resolution of acute edema and inflammation, partial healing of the extra-articular structures that may simplify the reconstruction, and the possibility of more detailed surgical planning.

Evidence on Early Versus Delayed Surgery

The risk of intra-articular injury associated with delayed surgery is supported by two meta-analyses of differing methodology. Analyzing 14 studies and 1,172 patients, Vermeijden et al. did not identify overall differences in complication rates, stiffness, mobility deficits, or general functional outcomes; however, the subgroup undergoing surgery before three weeks did show advantages, with a 30% lower incidence of meniscal injuries (RR 0.70; p = 0.04), a 50% lower incidence of chondral injuries (RR 0.50; p < 0.001), and Lysholm scores 6.8 points higher (p = 0.01) [13]. Kim et al. confirmed this pattern, quantifying the additional risk of delaying surgery; they found a higher incidence of meniscal injury (OR 1.73; 95% CI 1.10-2.73; p = 0.01) and chondral injury (OR 2.48; 95% CI 1.46-4.20; p = 0.0007), and lower Lysholm scores in the late surgery group [42]. Both meta-analyses point in the same direction for meniscal and chondral injury despite their methodological differences.

In a systematic review analyzing six comparative studies, Vicenti et al. found that surgery within the first three weeks was associated with higher mean Lysholm scores (89 versus 82), a greater proportion of excellent or good International Knee Documentation Committee (IKDC) results (57% versus 41%), and a better range of motion (129° versus 124°), concluding that intervention should preferably be performed within that interval [14]. In contrast, the systematic review by Marder et al., which included 31 studies and 2,594 patients, could not establish the superiority of any timing strategy, in part because of the great methodological heterogeneity of the studies and the impossibility of performing statistical comparisons between groups. The mean scores in the acute surgery group (Lysholm 73.60; IKDC 67.61; Tegner 5.06) were numerically lower than those in the delayed group (Lysholm 85.23; IKDC 72.32; Tegner 4.85), a difference the authors attribute in part to the greater injury severity of the first group and not necessarily to timing [12].

The prospective series by LaPrade et al., with 194 patients who underwent single-stage multiligament reconstruction, found no significant differences in functional outcomes between surgery performed in the acute and chronic phases, with clinically relevant improvements in all evaluated parameters regardless of the timing of surgery. Patients improved from a mean Lysholm score of 41 points preoperatively to 90 points at the end of follow-up, and functional activity measured by the Tegner scale rose from level 1 to level 6. Reoperation for arthrofibrosis was required in 9.3% of cases [43]. Zsidai et al. concluded that current evidence points to clinical equivalence or superiority of early over delayed treatment in most evaluated outcomes [41].

Clinical Situations That Modify Timing

Some clinical situations modify the standard timing algorithm. A vascular emergency requires immediate temporary stabilization with an external fixator followed by vascular repair, relegating ligament reconstruction to a second stage [6]. Polytrauma, periarticular fractures, soft-tissue compromise with blisters or open wounds, and the patient's general health are factors that may justify a staged approach. The Delphi consensus by Murray et al., with 39 international experts from 14 countries, agreed that single-stage surgery should be performed whenever possible (92.3% agreement) [2]. Despite the controversy and the scarcity of high-level studies, the literature reviewed here favors early intervention, within the first two to three weeks, once acute inflammation has resolved, and local conditions have been optimized.

Reconstruction techniques

General Principles

Anatomic reconstruction of all injured structures is the technical standard for the surgical management of MLKIs, as opposed to primary repair, which is reserved mainly for bony avulsions in patients with good-quality tissue and an early diagnosis [2,30,44]. The goal is to restore the knee's normal kinematics by reconstructing each structure anatomically and with appropriate tension. The choice between single-stage and staged surgery depends on the status of the soft tissues, the associated injuries, and the surgeon's experience, with a growing trend toward comprehensive anatomic reconstruction in a single intervention when technical conditions allow [3,30,45].

Reconstruction of the Cruciate Ligaments

Reconstruction of the PCL is central to most multiligament surgical techniques, given its role as the primary stabilizer of posterior tibial translation. Schreier et al. summarized the current principles: precise identification of the anatomic insertion points of the anterolateral and posteromedial bundles, preferential use of independent femoral tunnels to reproduce the bipolar architecture, and selection of grafts of sufficient length and caliber to withstand functional tension. The double-bundle technique, although technically more demanding, has shown more physiological biomechanical results in cadaveric studies compared with single-bundle reconstruction [24]. Therrien et al. also described the all-inside technique for PCL reconstruction, which minimizes tibial drilling and reduces the morbidity associated with the tibial tunnel, with clinical results comparable to conventional techniques at short- and medium-term follow-up [25].

Simultaneous reconstruction of the ACL and PCL, described in detail by Tang and Zhao, requires rigorous planning of the tunnel trajectories to avoid convergence; the usual order of tensioning and fixation (PCL anterolateral, PCL posteromedial, ACL, PLC, and PMC) preserves the central position of the pivot during the remainder of the reconstruction [46].

Reconstruction of the Collateral Complexes

Anatomic reconstruction of the PLC has undergone considerable technical evolution over the past decade. Maheshwer et al. describe the reference anatomic technique: combined reconstruction of the LCL, the popliteus tendon, and the popliteofibular ligament using independent grafts and differentiated femoral, fibular, and tibial tunnels [47]. The systematic review by Colatruglio et al. focused on the comparison between tibial-based and fibular-based techniques, found no significant clinical differences in medium-term outcomes, although the complete anatomic technique has a theoretical advantage in restoring external rotation [48]. Isolated repair of the PLC shows significantly higher failure rates than reconstruction and should be reserved for selected cases with bony avulsions and an acute diagnosis [47].

On the medial side, anatomic reconstruction of the posteromedial complex integrates the superficial MCL, the posterior oblique ligament, and the posteromedial capsule. In a series with a minimum two-year follow-up, Tapasvi et al. showed that anatomic reconstruction of the medial complex restores valgus stability and rotational kinematics, with good functional scores [26]. Marcheggiani Muccioli et al. described a minimally invasive double-bundle technique for the PMC without tibial tunnels, which reduces the risk of convergence with the tibial tunnels of the ACL and PCL [49]. In a multicenter study, Gensior et al. validated the role of augmented anatomic repair for acute combined PCL injuries with posteromedial or PLC involvement, with promising short-term functional results [50].

Technical Considerations: Tunnel Sequence and Convergence

Three-dimensional planning of the bone tunnels is a critical technical aspect of multiligament surgery. The anatomic proximity of the insertion points of the ACL, PCL, medial complex, and lateral complex generates a high risk of convergence, which can compromise fixation and graft integrity. Nassar et al. systematized the current strategies to minimize this risk: divergent angulation of the drills in the sagittal and axial planes, use of short tunnels when technically possible, preoperative planning with CT, and sequential execution of the tunnels in an order that allows intraoperative adjustments [51]. The fixation sequence recommended in most current protocols preserves reconstruction of the central pivot (PCL) first, before tensioning the peripheral structures, which avoids secondary displacement of the cruciate grafts [30,45].

Graft selection

Autograft Versus Allograft: Overview

The debate over which graft to use remains open, and the decision depends on the patient, the structure to be reconstructed, and the available tissue. The theoretical advantages of autograft are better biological integration, the absence of immunological risk, and lower cost, whereas its disadvantages include donor-site morbidity, increased surgical time, and quantitative limitation of tissue when several structures must be reconstructed simultaneously [2,3]. Allograft offers availability that is not limited by the size or number of grafts required, the absence of donor morbidity, and shorter operative time, but it carries a higher cost, a theoretical risk of infectious transmission, slower biological integration, and, in young, active patients, higher failure rates [2]. In the specific context of MLKIs, where multiple grafts are required in a single intervention, the combination of autograft and allograft in the same patient is a common practice.

Structure-Specific Evidence

The available evidence on the optimal graft choice differs according to the structure considered. In isolated ACL reconstruction in young, active patients, autograft has consistently shown lower failure rates than allograft, which is why it is considered the standard in this population group [2]. In the case of the PCL, the available evidence shows comparable results between the two options in terms of function and stability, although doubts persist about the long-term durability of allograft in young patients [24].

In a comparative retrospective study of 69 patients undergoing LCL reconstruction concomitant with ACL reconstruction, Dekker et al. found no significant differences between autograft (semitendinosus, 50 patients) and allograft (19 patients) in terms of varus stability, IKDC, Tegner, Lysholm, or patient satisfaction at a mean follow-up of 3.6 years [52]. For LCL reconstruction, therefore, the two graft sources performed alike on every outcome measured.

The systematic review and meta-analysis by Kern et al. on PLC reconstruction supports this conclusion: autograft and allograft show equivalent failure rates, although Lysholm scores are significantly higher (p = 0.04) in patients treated with autograft. The authors conclude that the final decision should be individualized according to age, activity level, and the availability of autologous tissue, and acknowledge that the methodological quality of the available studies remains limited [53].

For the MCL complex and the PMC, direct comparative evidence is scarce. The described anatomic techniques generally use autograft when the available tissue is sufficient, since they require grafts of moderate length that can be obtained from the semitendinosus or gracilis tendon [26].

Autograft Options and Donor-Site Morbidity

The choice of the specific autograft is an additional decision when autologous tissue is selected. The usual sources include the patellar tendon (bone-tendon-bone), the hamstring tendons (semitendinosus and gracilis), the quadriceps tendon, and, more recently, the peroneus longus tendon and the rectus femoris tendon. The choice depends on the required caliber and length, the planned concomitant surgery (for example, simultaneous ACL and PCL reconstruction), and the surgeon's preferences regarding donor-site morbidity.

Franciozi et al. described the technique for harvesting the full peroneus longus tendon graft, whose length, caliber, and limited functional morbidity at the ankle suit the multiligament setting [54]. It is an option when the hamstring autografts have already been harvested, since it avoids resorting to allograft for peripheral structures. Donor-site morbidity of the peroneus longus is generally low at medium-term follow-up, although comparative evidence against other autologous sources remains limited.

The rectus femoris tendon, the superficial layer of the quadriceps tendon, is the most recent addition. Stripped along a natural cleavage plane without a bone block, it preserves the deeper quadriceps layers and is long enough for double, triple, or four-strand configurations [55]. Doubled, its load to failure falls within the range of conventional ACL grafts, although its greater elongation raises questions about late laxity [55,56]. Early series report outcomes equivalent to hamstring autograft with low donor-site morbidity, and in the multiligament setting it spares the hamstrings and reduces dependence on allograft; the evidence, however, remains level III and IV with short follow-up [55,56].

There is no universally superior graft. The available data support functional equivalence between autograft and allograft for PLC and PCL reconstruction, whereas autograft remains preferred for the ACL in young, active patients [2,52,53]. Table 2 summarizes the graft of choice and the evidence behind it for each structure.

**Table 2:** Graft selection by structure in multiligament knee injuries ACL: anterior cruciate ligament; PCL: posterior cruciate ligament; LCL: lateral collateral ligament; PLC: posterolateral corner; MCL: medial collateral ligament; PMC: posteromedial corner; IKDC: International Knee Documentation Committee The autograft sources listed are those described in the text as suitable for each structure; no study has compared them head-to-head for a given structure. Adapted from Murray et al., Schreier et al., Dekker et al., Kern et al., Franciozi et al., Barroso et al., Glaab et al., and the anatomic technique descriptions cited in the text.

Structure	Graft of choice	Evidence and comment
ACL	Autograft in young, active patients: bone-patellar tendon-bone, hamstring (semitendinosus-gracilis), quadriceps tendon, or the rectus femoris tendon as an emerging alternative	Consistently lower failure rates than allograft, which makes autograft the standard in this population [2]
PCL	Autograft or allograft; as autograft, hamstring or quadriceps tendon chosen by the caliber and length the technique requires, with the rectus femoris tendon described for double-bundle reconstruction	Comparable function and stability between sources; doubts persist about long-term allograft durability in young patients [24]
LCL	Autograft or allograft; semitendinosus is the autograft used in the comparative series	Dekker et al., 69 patients: no differences in varus stability, IKDC, Tegner, Lysholm, or satisfaction at 3.6 years [52]
PLC	Autograft when tissue is available, usually semitendinosus, or peroneus longus if the hamstrings have already been harvested; allograft equally acceptable	Kern et al.: equivalent failure rates, higher Lysholm scores with autograft (p = 0.04). Peroneus longus avoids resorting to allograft for peripheral structures [53,54]
MCL and PMC	Autograft, semitendinosus or gracilis	Scarce direct comparative evidence; the described anatomic techniques need grafts of moderate length, which these tendons provide [26]

Rehabilitation and return to sport

Principles and Phases of Postoperative Rehabilitation

Postoperative rehabilitation after multiligament surgery influences the final functional outcome, together with the quality of the surgical technique and the initial injury pattern. Protocols have shifted from the prolonged immobilization that characterized the classic series toward regimens of early controlled mobilization, which typically begin within the first 24 to 48 postoperative hours and are associated with a lower incidence of residual stiffness without compromising graft integrity [2,30,57].

Nielsen et al. described the recovery phases after multiligament reconstruction in four sequential stages: a first stage of protection and restoration of the range of motion (weeks 0 to 6), followed by a phase oriented toward the recovery of muscle strength and neuromuscular control (weeks 6 to 10). A phase of progressive reintegration into functional activity then begins (from week 10 to the sixth postoperative month), and the final stage, aimed at return to sport and specific training, usually extends between the sixth and twelfth month after surgery. Progression through each phase is based on objective criteria (range of motion, isokinetic strength, symmetry with the contralateral limb) rather than on rigid chronological timelines [58].

Monson et al. described the postoperative protocol in detail, with emphasis on early quadriceps activation, the use of dynamic braces adapted to the injury pattern, particularly in PCL-based injuries, and the gradual progression of weight-bearing [57].

The controversy about the optimal time to begin intensive rehabilitation was addressed by Hoit et al. in the only randomized clinical trial available in this field. In a pilot study of 36 patients with MLKIs that compared early rehabilitation (beginning on the first postoperative day) with delayed rehabilitation (three weeks of immobilization followed by the same protocol), the authors found no significant differences in the rate of manipulation under anesthesia, postoperative laxity, or patient-reported outcomes at one year of follow-up [59]. These results suggest that early rehabilitation is safe and can be considered the option of choice when the status of the soft tissues and the quality of fixation allow.

Return to Sport and Activity

The systematic review by D'Ambrosi et al. synthesized the available data and reported that approximately 75% of patients who undergo multiligament reconstruction return to some form of sports practice, although only around 60% do so at their pre-injury level. The main predictors of return include young age, the previous sports level, the injury pattern (with worse results in PCL-based injuries), and the absence of postoperative complications [60].

In the subgroup of young athletes, Fine et al. reported higher return rates than in the overall MLKI population, although a gap persists between returning to any level of sport and returning to the previous competitive level. Return to competitive sport typically requires a minimum period of nine to 12 postoperative months, conditioned by the achievement of objective functional tests and by the clinical decision of the treating team [57,61]. Consistent with this, Dean et al. reported significantly higher postoperative activity scores after low-energy than after high-energy injuries [23].

Long-term functional outcomes

Overall Outcomes and Deterioration Over Time

Functional outcomes after multiligament surgery are acceptable in the short term but tend to be unfavorable with long-term follow-up. The meta-analysis by Klasan et al. quantified this temporal evolution. In the first two years, patients recovered between 80% and 85% of their pre-injury function, but from then on the IKDC score decreases at a rate of almost two points per year (-1.99 points/year), and the Lysholm score declines continuously as well, although more gradually (-0.80 points/year). The deterioration is especially pronounced when the injury is centered on the PCL, a finding that points to the posterior stabilizer as a determinant of long-term function [16].

Moews et al. reviewed studies with seven or more years of follow-up (16 studies, 730 knees) and found that the long-term prognosis is less favorable than the two-year results suggested. High-grade radiographic osteoarthritis (Kellgren-Lawrence 3-4) affected between 12% and 71.7% of patients depending on the series, a range that reflects the heterogeneity in injury profiles and surgical techniques. Reoperations, mainly for stiffness, ranged from 1.7% to 34.3%, and up to 10.9% of patients in some cohorts ultimately required a total knee arthroplasty [17].

Specific Series and Prognostic Factors

In a series of 20 knees with a mean follow-up of 13.1 years, Zhang et al. reported acceptable functional scores but a high prevalence of radiographic degenerative changes [62]. Focusing specifically on PCL-based MLKIs with PMC involvement at 10 years of follow-up, Pizza et al. identified PMC rupture as an independent risk factor for reconstruction failure and described acceptable functional survivorship, with good return-to-work and return-to-recreational-sport rates in patients who avoided major complications [63]. Djebara and Pujol provided parallel data on MLKIs with lateral involvement at 7.5 years, with overall good functional results, although conditioned by the complexity of the initial injury pattern [64].

Clinical Implications

Multiligament surgery restores acceptable function in most patients, but that recovery is not definitive: progressive deterioration and post-traumatic osteoarthritis are expected over the long term, particularly in PCL-based injuries. The prognostic factors that drive this course, namely the injury mechanism, the structural pattern, associated meniscal and chondral injuries, age, and the timing of surgery, belong in the preoperative conversation with the patient, since they set what can realistically be expected [16,17,36].

Complications

Arthrofibrosis and Postoperative Stiffness

Although arthrofibrosis appears in almost all MLKI series, its definition remains heterogeneous. Fahlbusch et al. proposed as a diagnostic threshold an extension deficit greater than 10° or a flexion deficit greater than 25° compared with the contralateral limb, a criterion used by only 12% of the studies they reviewed. This lack of standardization partly explains the wide dispersion of the prevalence reported in the literature, from 2.8% to 57.1% depending on the series. Across 25 studies and 709 patients, the overall prevalence was 12.1%. Eighty-eight percent of the analyzed publications use subjective definitions, so uniform operational criteria are needed to allow results to be compared [15].

The main risk factors identified are the severity of the injury pattern, particularly Schenck KD III and KD IV injuries, surgical treatment in the acute phase, and restrictive rehabilitation protocols in the first three months [15]. Feingold et al., studying KD III and KD IV injuries, reported a significantly higher incidence (32.4%) than in the overall MLKI population, which supports the initial injury pattern as an independent predictor [65]. The multicenter study by Bi et al., which analyzed 190 patients treated at two academic centers over 19 years, found that body mass index, even in grades of morbid obesity, is not significantly associated with a higher risk of postoperative stiffness, whereas the use of external fixation at the time of the initial surgery (OR 3.3) and the presence of associated vascular injury (OR 6.2) are independent predictors of the need for manipulation under anesthesia [66].

The therapeutic management of arthrofibrosis includes intensive physiotherapy in the early stages and, in refractory cases, manipulation under anesthesia with or without arthroscopic release of adhesions, a procedure typically performed at three to six months postoperatively. Active research seeks pharmacological alternatives to reduce the incidence: the LION (Losartan to Improve Outcomes after multi-ligament kNee injury) trial, described by Kehoe et al., is the first multicenter randomized clinical trial to evaluate losartan as a pharmacological agent to reduce arthrofibrosis after multiligament surgery, supported by the antifibrotic activity of angiotensin II receptor antagonists demonstrated in preclinical models. Its results are expected in the coming years [67].

Residual Instability and Graft Failure

Residual instability is the second most frequent complication and the main cause of surgical reintervention after multiligament surgery. Its origin is multifactorial and includes technical errors (non-anatomic tunnel positioning, inadequate graft tension, incorrect fixation sequence), insufficient graft quality, biological failure of integration, or early functional overload. Failure rates vary considerably according to the structure considered: PCL reconstruction shows a graft failure incidence between 5% and 15% at medium-term follow-up [24], whereas PLC reconstruction shows a failure incidence between 0% and 9.1% depending on the series [53].

The systematic review by Moews et al. reported reintervention rates for ligamentous causes between 1.7% and 34.3% at follow-up beyond seven years, with PCL reconstructions being the most frequently revised procedure [17]. Revision techniques are more demanding than primary surgery because of the presence of previous tunnels, alteration of the local anatomy, and frequently greater soft-tissue compromise. Preoperative planning with CT and careful consideration of graft options are decisive for the success of revision surgery [51].

Late Neurovascular Injury and Post-traumatic Osteoarthritis

Although neurovascular injury is usually diagnosed in the acute phase, complex regional pain syndrome, residual common peroneal nerve neuropathy, and, less frequently, late complications of vascular repair may present during follow-up. Functional recovery from a complete nerve injury is often incomplete and is a frequent cause of residual disability. Tendon transfer, usually of the tibialis posterior, is reserved for cases without recovery after prolonged observation [1,4].

Post-traumatic osteoarthritis is the longest-term complication and affects the patient's quality of life. Up to 71.7% of patients present with Kellgren-Lawrence grade 3-4 radiographic osteoarthritis at seven or more years after surgery, depending on the series analyzed [17]. Independent risk factors include associated meniscal and chondral injury, residual instability, and a high-energy injury mechanism. These risk factors are the rationale for precise anatomic reconstruction of all injured structures, concomitant treatment of repairable meniscal injuries, and rehabilitation aimed at avoiding both stiffness and residual instability, although no study has yet shown that this strategy reduces the incidence of osteoarthritis.

Complications after this surgery are frequent and can weigh on the final outcome as much as the technique itself: arthrofibrosis and residual instability account for most reinterventions, and post-traumatic osteoarthritis defines the long-term course. Identifying the risk factors described here before surgery is what allows them to be anticipated [2,15,65].

Discussion

The analyzed data confirm the initial premise of this review: surgery is the treatment of choice in most patients with MLKIs, although important controversies persist regarding the timing of intervention, graft selection, and the sequence of reconstruction. The international Delphi consensus by Murray et al., with 100% agreement among the experts consulted, identifies surgical treatment as the option of choice over conservative management in young patients [2], and both the prospective series by LaPrade et al. and the pooled data of Klasan et al. document substantial clinical improvement after multiligament reconstruction, although neither includes a conservatively managed comparison group [16,43]. The systematic review by Marder et al. could not establish the superiority of any timing strategy [12], the comparative evidence on grafts in peripheral structures is heterogeneous [52,53], and the optimal sequence of intraoperative tensioning and fixation continues to rely mostly on biomechanical studies and expert consensus, without comparative clinical trials.

Surgical Timing: Divergent Evidence

The most recent systematic reviews on timing reach divergent conclusions despite analyzing largely the same primary studies. The discrepancy does not reflect differences in the literature, but in the cutoff points adopted to define early surgery, so their pooled estimates cannot be read as independent results or aggregated with one another. The reviews that applied a three-week cutoff found fewer secondary meniscal and chondral injuries in the early group [13,42], whereas those pooling studies with heterogeneous cutoffs could not establish any superiority [12]. Vicenti et al. reported clinical differences favoring early treatment [14] and LaPrade et al. detected none attributable to timing [43]; no randomized trial has addressed the timing of surgery itself [59].

This heterogeneity is partly due to the absence of uniform definitions: the cutoff points for early surgery range from 48 hours to one year depending on the study considered [41], which limits the comparability of results. Added to this is a probable selection bias, since more severe injuries or those with vascular compromise tend to undergo surgery later, whereas less complex injuries allow early reconstruction. When this bias is attenuated, as occurs in analyses with a three-week cutoff, the trend points to clinical equivalence between the two strategies and to a potential benefit of early treatment in preventing secondary intra-articular injuries. The relative risks and odds ratios supporting that signal come from meta-analyses of observational studies, so they quantify association under the definitions used by each review and not a treatment effect established by randomization.

A reasonable approach, according to the available literature, is to favor early intervention, understood as that performed within the first two to three weeks after the injury, once acute inflammation has resolved and provided that the status of the soft tissues, hemodynamic stability, and local conditions allow. This trend is consistent with the Delphi consensus, which favors single-stage surgery when feasible (92.3% agreement) [2], and with the lower risk of arthrofibrosis reported with arthroscopic techniques and accelerated rehabilitation [41]. Recognizing this trend does not, however, mean ruling out delayed treatment in specific scenarios, particularly in the setting of a vascular emergency, polytrauma, or soft-tissue compromise.

Graft Selection and Reconstruction Technique

The debate about the type of graft has shifted in recent years from a choice between autograft and allograft toward an approach differentiated by injured structure and by patient profile. For the LCL and the PLC, neither Dekker et al. nor Kern et al. found differences in failure rates between autograft and allograft [52,53]. For the ACL, the evidence continues to favor autograft in young, athletically active patients, whereas for the PCL the results are comparable, with doubts about the long-term durability of allograft. The range of available autografts has widened in the same direction: the full peroneus longus tendon described by Franciozi et al. [54] and, more recently, the isolated rectus femoris tendon [55,56]. Both ease the shortage of tissue that constrains multiligament reconstruction and reduce dependence on allograft. Their evidence base, however, is not comparable to that of the established sources.

The reviewed evidence, observational in design, consistently favors anatomic reconstruction over primary repair for the collateral complexes. Isolated repair of the PLC fails more often and is therefore limited to acute bony avulsions [47]. On the medial side, anatomic reconstruction of the posteromedial complex offers consistent results [26], whereas augmented repair, described by Gensior et al., is a reasonable alternative in acute injuries combined with the PCL, without displacing reconstruction as the technique of choice [50]. Complete anatomic reconstruction of all injured structures should therefore be prioritized, with repair reserved for well-defined scenarios.

Classification and Prognostic Value

The findings of Green et al. and of Sanchez-Munoz et al. challenge some assumptions surrounding the Schenck classification. The rarity of a radiographically documented dislocation among cases published as KD I casts doubt on the validity of this category and supports a subclassification or redefinition [34,35]. The inability of the Schenck grade to predict associated vascular injury [11], together with its limited discriminative capacity relative to other prognostic factors [36], suggests that the system, despite its usefulness as a clinical communication tool, should not be interpreted as a prognostic predictor per se. Meniscal, chondral, and neurovascular injuries and the dislocation mechanism determine the prognosis at least as much as the KD grade does.

Outcomes Over Time

Functional outcomes after multiligament reconstruction show an unfavorable trajectory over time. Klasan et al. quantified the continuous decline in IKDC and Lysholm scores, most marked in injuries involving the PCL [16], and Moews et al. confirmed the same progression over the very long term [17]. Multiligament surgery remains the option supported by the available evidence, although the published series include no conservatively managed comparison group; the progressive functional deterioration should be stated explicitly in the informed consent process.

Limitations and Research Priorities

This work has the limitations expected of a narrative review. The search was purposive rather than exhaustive, record-level screening counts were not tabulated, study selection and data extraction were carried out by a single author without duplicate independent extraction, and no formal risk-of-bias tool was applied to the included studies, so the appraisal of their quality rests on study design alone. Methodological heterogeneity limits what can be concluded from the pooled literature. The definitions of arthrofibrosis and the thresholds for early surgery and graft failure vary widely across series [15]. The available evidence is dominated by retrospective series and systematic reviews of observational studies, with an absence of randomized clinical trials on timing, reconstruction sequence, and graft selection. The only identified trial (Hoit et al.) specifically addresses the timing of rehabilitation onset and not the surgical decision itself [59]. Finally, no specific Cochrane review on MLKIs that synthesizes this literature with a standardized methodology is available, which is a relevant gap in the evidence.

Two gaps define the priorities for future research: the absence of comparative clinical trials on timing and grafts, and the lack of uniform definitions for the main complications. The LION trial of losartan as an antifibrotic agent could modify the approach to arthrofibrosis [67]. The development of all-inside techniques for cruciate ligament reconstruction, the consolidation of anatomic techniques for the collateral complexes with a lower risk of tunnel convergence [49,51], and the widening range of available autografts [54-56] are areas of ongoing technical development. The immediate tasks are to agree on outcome definitions in prospective multicenter registries and to design trials that compare timing and graft strategies directly; until then, recommendations will continue to rest largely on expert consensus.

## Conclusions

MLKIs are uncommon but limb-threatening, and their management depends on a systematic assessment of every injured structure. In active patients, surgery is the treatment of choice, and all injured structures should be reconstructed anatomically, ideally in a single stage within the first two to three weeks, because delay beyond this window is associated with more secondary meniscal and chondral damage in observational series. The Schenck grade is useful to describe the injury but should not be used on its own to predict prognosis or neurovascular risk: every patient needs a systematic vascular assessment, with a low threshold for CT angiography and particular vigilance when the PLC is involved, since associated neurovascular injury is the complication in which delay determines whether the limb is saved. Graft choice should follow the structure and the patient, with autograft preferred for the ACL in young, active patients and autograft or allograft used interchangeably for the PCL and the collateral complexes. Early controlled mobilization should be the default, as it is associated with a lower risk of arthrofibrosis without compromising the reconstruction.

What the surgeon conveys before surgery matters just as much. Multiligament reconstruction restores acceptable function in the short and medium term, but results decline progressively beyond about seven years, with post-traumatic osteoarthritis and reintervention for stiffness that are more marked in PCL-based injuries, so preoperative counseling should set realistic expectations. Three questions remain open: the optimal timing of surgery, graft selection, and the sequence of reconstruction. All three still rest largely on expert consensus, and resolving them will require comparative trials with uniform outcome definitions and prospective multicenter registries. Until then, comprehensive anatomic reconstruction, systematic neurovascular assessment, early rehabilitation, and honest preoperative counseling remain the standard approach.
